# scTree: An R package to generate antibody-compatible classifiers from single-cell sequencing data

**DOI:** 10.21105/joss.02061

**Published:** 2020-04-26

**Authors:** J. Sebastian Paez, Michael K. Wendt, Nadia Atallah Lanman

**Affiliations:** 1 Purdue University, Center for Cancer Research; 2 Purdue University, Department of Medicinal Chemistry and Molecular Pharmacology; 3 Purdue University, Department of Comparative Pathobiology

## Abstract

Single-cell RNA sequencing (scRNA-seq) is now a commonly used technique to measure the transcriptome of populations of cells. Clustering heterogeneous cells based on these transcriptomes enables identification of cell populations (Butler, Hoffman, Smibert, Papalexi, & Satija, 2018; Trapnell et al., 2014). There are multiple methods available to identify “marker” genes that differ between these populations (Butler et al., 2018; Love, Huber, & Anders, 2014; Robinson, McCarthy, & Smyth, 2009). However, there are usually too many genes in these lists to directly suggest an experimental follow-up strategy for selecting them from a bulk population (e.g. via FACS (Tung et al., 2007)). Here we present scTree, a tool that aims to provide biologists using the R programming language and scRNA-seq analysis programs a minimal set of genes that can be used in downstream experiments. The package is free, open source and available though GitHub at github.com/jspaezp/sctree

## Implementation and results

The underlying model behind scTree is a combination of random forest for variable selection and a classification tree; having this model as a classifier relies on the fact that classification trees are analogous to many approaches in biology such as the gating strategy employed in flow cytometry or Fluorescence assisted cell sorting (FACS) experiments. In flow cytometry and FACS experiments, populations are identified and sorted based on expression levels of distinct markers that entail the identity or state of the chosen population. Usually such experiments use only relative levels of marker expression, using terms such as “High” and “Low” (Coquery, Loo, Buszko, Lannigan, & Erickson, 2012; Robertson & Scadden, 2005).

In a similar manner, scTree produces accurate, biologically relevant, and easily interpretable results, which can be used for subsequent subpopulation sorting and biological validation by fitting shallow decision trees analogous to FACS sorting strategies and is able to output these classifiers in a format easily interpretable in a wet-lab setting.

The method to calculate variable importances based on random forests has been previously described, and has been implemented in R by the ranger package (Altmann, Toloşi, Sander, & Lengauer, 2010; Janitza, Celik, & Boulesteix, 2018; Wright & Ziegler, 2017). The suggestion of gating strategies is achieved by fitting a classification tree using the implementation provided by the partykit R package (Hothorn & Zeileis, 2015).

In order to benchmark the quality of markers, we utilized a recall-based strategy. Briefly, each dataset was split randomly into two sections, a training set with 80% of the cells and a testing set consisting of the 20% remaining. A classifier was trained by selecting the top 5 markers suggested for each cluster by either scTree (Altman method) or by two of the most commonly used marker gene detection methods for scRNA-seq data: t-tests or wilcoxon-tests (as implemented by Seuratv3.0.1).

**Figure 1:**
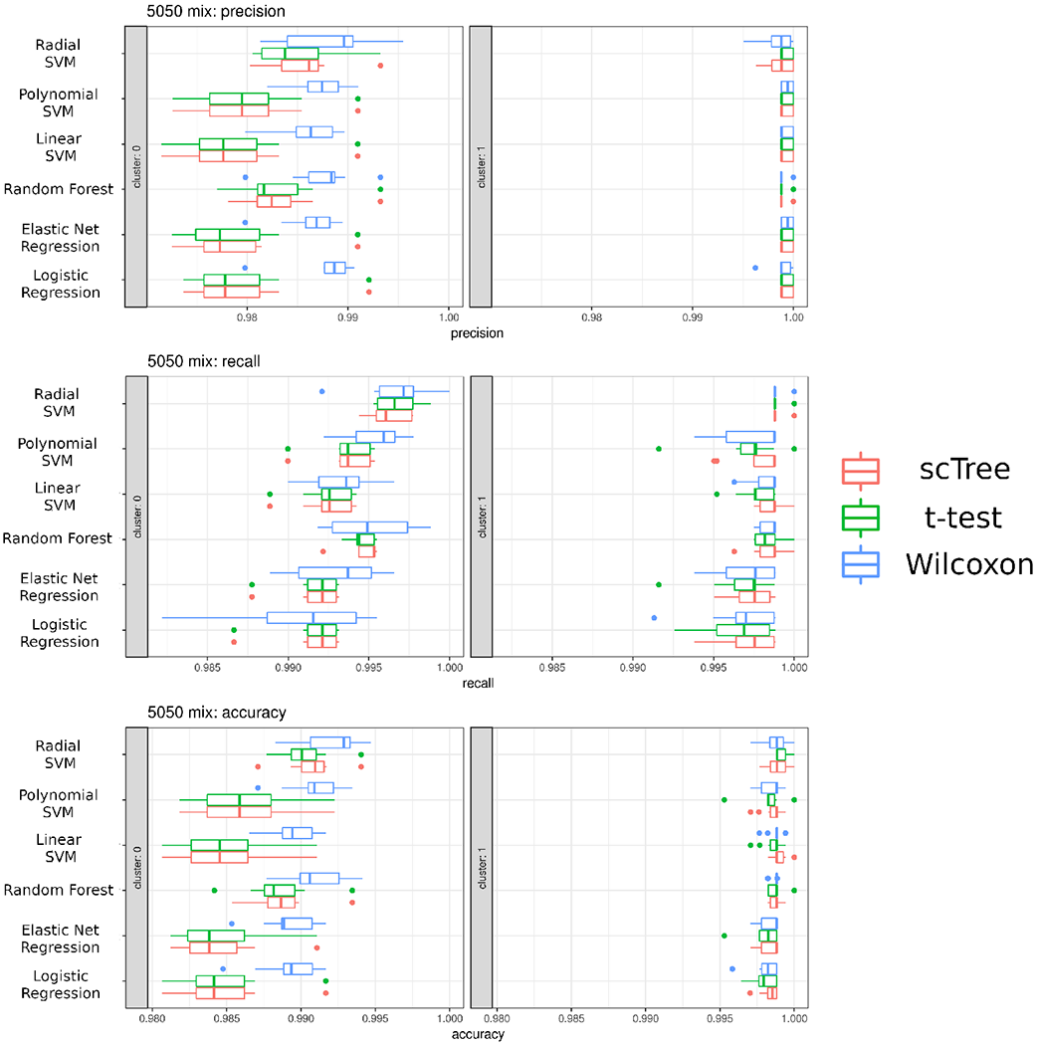
Depiction of the classification performance achieved in the Jurkat:293 50:50 dataset. A number of machine learning algorithms were tested to ensure that scTree performed as well as traditional marker identification approaches, regardless of the classifier used.

These classifiers were then used to predict the identity of the testing set and the quality was assesed by comparing the recall, accuracy and precision of the prediction. We were concerned that the forest-based markers would artificially favor scTree, therefore we utilized several classifiers for the markers derived from either scTree, t-tests or wilcoxon-tests. As shown in Figures 1 **and** 2, bias was not observed, and regardless of the final classification model, the features selected by using scTree provide a comparable accuracy, precision and recall to those acquired using traditional differential expression methods. It is important to note that many of the wrongly assigned labels happen between cell populations that are hard to define in-vivo and are not resolved clusters in the UMAP dimensional reduction, such as macrophage subtypes and between NK and Tc cells.

### Example Output from the package

#### Predictor generation

**Figure 3:**
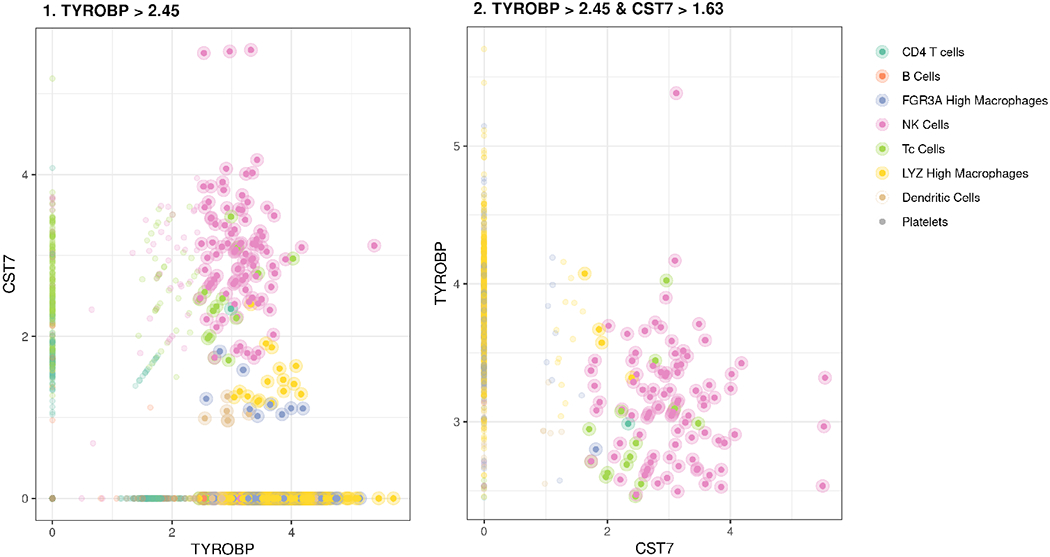
Scatterplot showing the progressive gating that would be used to classify node 11 in the 3K PBMC dataset. Filtering in each pane is done on the gene presented on the X-axis of the plot and cells kept during that filtering step are highlighted

As mentioned previously, a main focus in the development of scTree was the biological interepretability of the models. Therefore the models can be expressed as a Garnett file, as shown in Code Section 1, as specified originally in the Garnett manuscript by the Trapell lab (Pliner, Shendure, & Trapnell, 2019). Visualizations are designed to resemble flow cytometry results, as show in Figure 3 and connections with several antibody vendors are provided to query the availability of probes for the genes found to be usefull for classification.

**Code Section 1.**
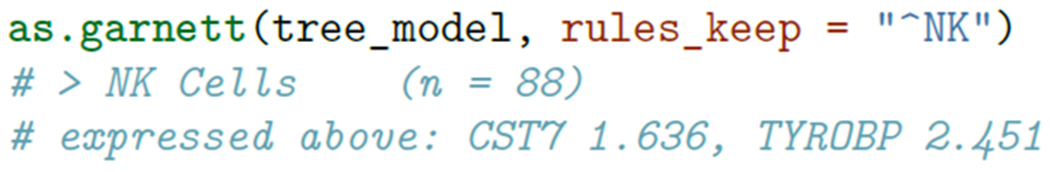
Suggested classification scheme for NK cell cluster of the PBMC dataset. The data depicts how the cluster corresponding to NK cells can be predominantly identified as GNLY High/GZMB High.

Despite scTree being originally developed for single cell sequencing, we recognize it could also be used for other high content single-cell workflows, such as CyTOF or data driven multiple-channel flow cytometry.

#### Antibody querying interface

The provided interface with antibody databases, further enhances the utility of scTree by fulfilling the need to interface *in silico* models and data with *in vitro* followup. Therefore, a package interface with common antibody vendors and search engines are provided. This interface is exemplified in Code section 2.

**Code Section 2.**
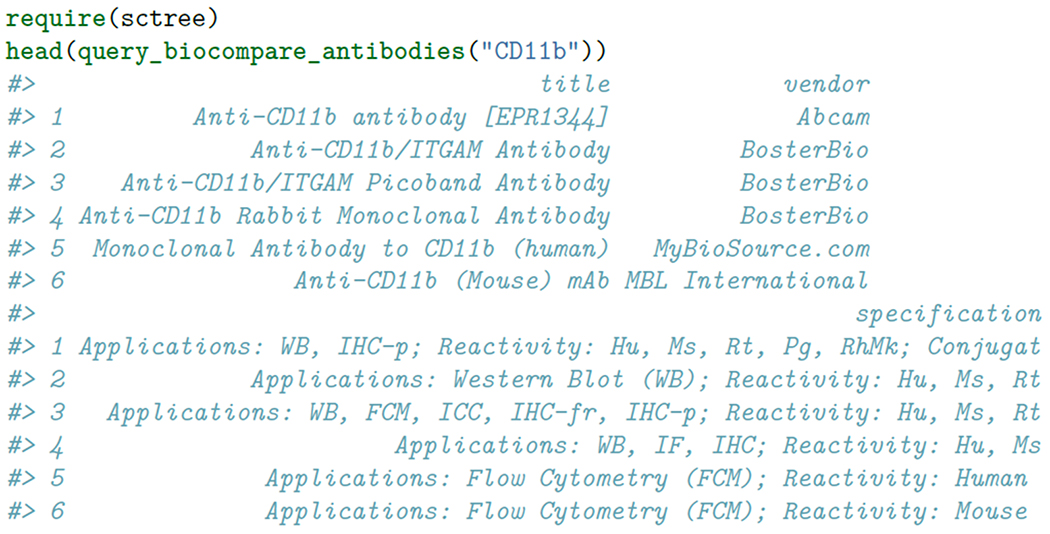
Example of the automated antibody query interface

Additional usage cases and up-to-date code snippets of the common functions can be found in the package documentation website (jspaezp.github.io/sctree/) and the readme file hosted in the github repository (github.com/jspaezp/sctree).

## Methods

### Testing dataset processing

The filtered raw counts for each dataset were downloaded from the 10× website single cell expression datasets (10X-Genomics, 2019) and were processed by the standard Seurat work-flow, as described in the package tutorial (“Satija-Lab, 2019”). This process was carried out for the following datasets:

1. 3k PBMC, Peripheral Blood Mononuclear Cells (PBMC)
2. 50%:50% Jurkat:293T Cell Mixture, originally published by Wan, H. et al. in 2017

### Description of the benchmarking process

Briefly, each dataset was split into a testing and a training set. For each cluster, each of the different marker identification methodologies was used and the top five markers were selected. These five markers were used to train a prediction model on the training set and the predicitons were carried out on the testing set. These predictions were compared with the assigned cluster identity and performance metrics were calculated.

### Formulas defining the prediction quality

$$precision=\frac{True\;Positives}{True\;Positives+False\;Positives}$$

$$recall=\frac{True\;Positives}{True\;Positives+False\;Negatives}$$

$$accuracy=\frac{True\;Positives+True\;Negatives}{Total}$$

## Figures and Tables

**Figure 2:**
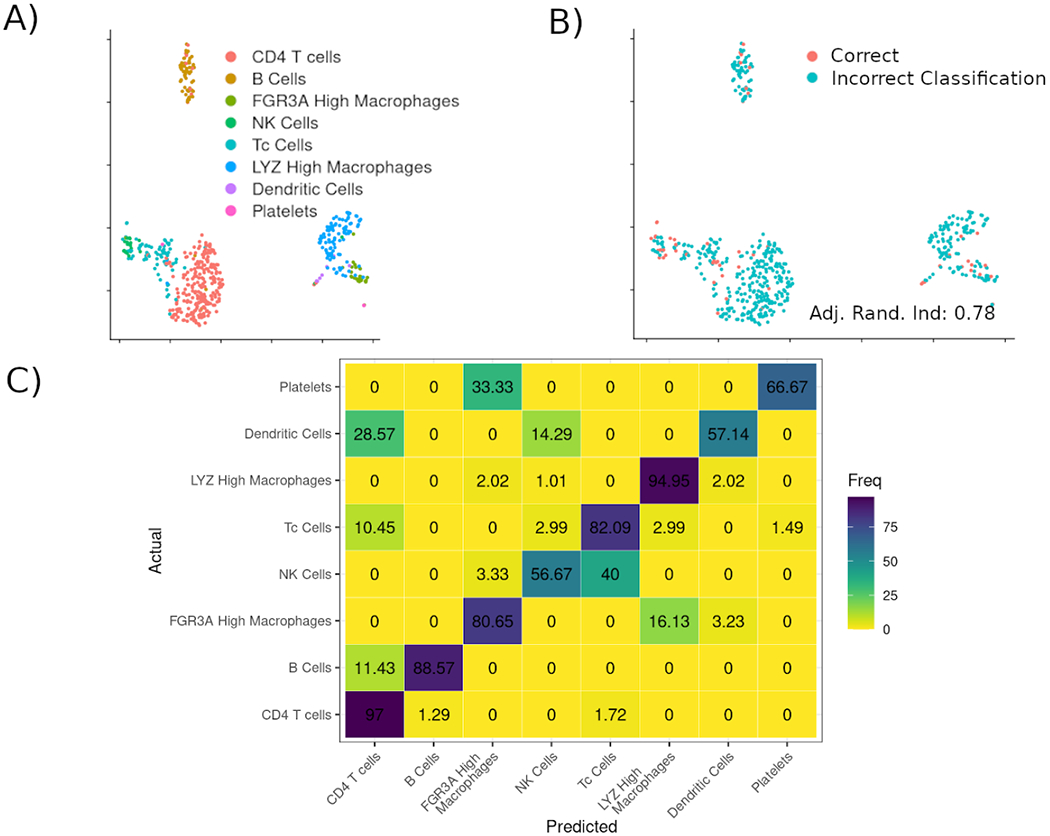
Depiction of the predicted identities in the PBMC 3k dataset dataset. **A.** Real identities are the identities as identified through unsupervised clustering performed using Seurat and annotated based on prior knowledge (Butler et al., 2018). **B.** The scTree package was then used to classify cells based on the top 5 markers for each cluster chosen by scTree and accurately recapitulates the original classification determined by Seurat. **C.** Confusion matrix showing the assigned classification to each cluster.
